# Supplementary material for: Global hypo-methylation in a proportion of glioblastoma enriched for an astrocytic signature is associated with increased invasion and altered immune landscape
Source: eLife. 2022 Nov 22;11:e77335. doi: 10.7554/eLife.77335 (PMC9681209; doi:10.7554/eLife.77335)
Supplement: Figure 2—source data 1. [file elife-77335-fig2-data1.zip › Figure_2_source_data_1/Figure_2I_J/homerResults/motif8.similar.html]

motif8

## Information for motif8

T
C
G
A
T
A
G
C
C
T
G
A
T
C
A
G
C
T
G
A
T
C
A
G
T
C
G
A
C
G
T
A
C
T
A
G
C
T
A
G
  
Reverse Opposite:  

G
A
T
C
G
A
T
C
G
C
A
T
A
G
C
T
A
G
T
C
G
A
C
T
A
G
T
C
G
A
C
T
A
T
C
G
A
G
C
T
  

|  |  |
| --- | --- |
| p-value: | 1e-44 |
| log p-value: | -1.026e+02 |
| Information Content per bp: | 1.680 |
| Number of Target Sequences with motif | 62.0 |
| Percentage of Target Sequences with motif | 8.54% |
| Number of Background Sequences with motif | 2.1 |
| Percentage of Background Sequences with motif | 0.75% |
| Average Position of motif in Targets | 111.9 +/- 55.5bp |
| Average Position of motif in Background | 125.4 +/- 50.3bp |
| Strand Bias (log2 ratio + to - strand density) | -0.6 |
| Multiplicity (# of sites on avg that occur together) | 1.10 |
| Motif File: | file (matrix) reverse opposite |

### Similar de novo motifs found

|  |  |  |  |  |  |  |  |
| --- | --- | --- | --- | --- | --- | --- | --- |
| Rank | Match Score | Redundant Motif | P-value | log P-value | % of Targets | % of Background | Motif file |
| 1 | 0.608 | A C G T A G T C C G A T G A T C C G A T C A G T A C G T A C G T A G T C G C T A | 1e-36 | -84.508789 | 5.92% | 0.70% | motif file (matrix) |
| 2 | 0.665 | C T G A C A T G T C G A T C A G T C G A T C G A C A T G T C A G T A C G C G A T C A T G T C A G C A T G G T A C G A C T | 1e-33 | -77.866165 | 9.50% | 1.64% | motif file (matrix) |
| 3 | 0.633 | G T C A G C A T G A T C G A C T A T G C G A T C G A C T A C G T A T G C G A C T | 1e-20 | -46.442285 | 5.23% | 0.80% | motif file (matrix) |
| 4 | 0.767 | G A T C G A C T A G C T A G T C G A T C G A C T A G C T A T G C G A C T A G T C G A C T A T G C C G A T A C T G C A G T | 1e-18 | -42.374263 | 4.96% | 0.94% | motif file (matrix) |
| 5 | 0.711 | C T G A T C G A T C G A C G T A C G T A T C A G C T G A A C T G T G C A C T G A T C A G C T G A | 1e-15 | -35.089788 | 5.37% | 1.29% | motif file (matrix) |
| 6 | 0.776 | A G T C A C G T A G T C A C G T A C T G A C G T | 1e-11 | -26.805922 | 18.46% | 10.06% | motif file (matrix) |
